# Hepatoprotective effect of lotus leaf against non-alcoholic fatty liver disease in rats via alteration of AMPK/SIRT1 and Nrf2/HO-1 signaling pathway

**DOI:** 10.1590/acb407025

**Published:** 2025-08-25

**Authors:** Qingxia Shen, Junqian Wang, Na Yao, Xiyan Niu, Mi Liu, Xiaohui Li

**Affiliations:** 1 Hebei Provincial Hospital of Chinese Medicine – Department of Hepatobiliary Medicine – Shijiazhuang – China.; 2 Hebei Provincial Hospital of Chinese Medicine – Department of Respiratory Medicine II – Shijiazhuang – China.; 3 Hebei Provincial Hospital of Chinese Medicine – Department of Tumor I – Shijiazhuang – China.

**Keywords:** Oxidative Stress, Hepatocytes, NF-kappa B, Inflammation

## Abstract

**Purpose:**

In this study, we scrutinized the protective effect of lotus leaf (LF) against high-fat diet (HFD) induced liver injury in rats.

**Methods:**

The rats received the HFD for the induction of non-alcoholic fatty liver disease. Rats received the oral administration of LF (25, 50, and 100 mg/kg, b.w.). The insulin level, organ index, glucose level, hepatic, oxidative stress, lipid and cytokines parameters were measured. The different mRNA expression and histopathology were performed in the hepatic tissue.

**Results:**

LF treatment suppressed the insulin, glucose and HOMA-IR along with organ index (liver index and spleen index). LF treatment altered the level of liver parameters (aspartate aminotransferase, alanine aminotransferase, alkaline phosphatase, gamma-glutamyl transferase) and oxidative stress parameters in the serum, as well as the liver tissue. LF treatment altered the level of lipid parameters and fat parameters (total fat, perirenal fat, abdominal fat, epididymal fat); cytokines (tumor necrosis factor-α, interleukin-1β, interleukin-6, interleukin-10, interleukin-17, interleukin-33); HO-1, and Nrf_2_. LF treatment altered the mRNA expression of tumor necrosis factor-α, interleukin-1β, interleukin-6, interleukin-10, caspase-3, caspase-9, cytochrome C, cytochrome D, AMP-activated protein kinase (AMPK), sirtuin 1 (SIRT1), FRX-1, liver X Receptor alpha, fibronectin, matrix metalloproteinase-9, inducible nitric oxide synthase, and transforming growth factor-β 1 (TGF-β1). LF treatment suppressed the necrosis of hepatocytes with less inflammatory cell infiltration in the liver tissue along with alteration of liver injury score.

**Conclusion:**

The result showed the protective effect of LF against non-alcoholic fatty liver disease via activating the AMPK/SIRT1 and Nrf_2_/HO-1 pathway activation.

## Introduction

Nonalcoholic fatty liver disease (NAFLD) is the exaggerated hepatocytic lipid deposition that is intimately correlated with obesity and type-II diabetes, and has become an emerging health challenge^1^,^2^. It is also related to the body fat accumulation, oxidative stress, and dyslipidemia. Previous report suggested that the patients suffer from NAFLD are characterized with body fat accumulation and dyslipidemia. The higher amount of lipid deposition in the liver can induce the excessive oxidation, fibrosis, and inflammation and enhance the progression from steatosis to non-alcoholic steatohepatitis (NASH)3. NASH cases can deteriorate the fibrotic liver and may turn into hepatocellular carcinoma (HCC), liver failure and cirrhosis in 20% of them^3^,^4^. Furthermore, there is currently no recognized medical treatment for NASH other than liver transplantation. Report suggested that liver fibrosis is one of the pathogeneses of NASH and its pathological process of the transition from chronic liver disease to cirrhosis.

Additionally, hypoxia inducible factor-1α (HIF-1α), which is one of the targets of hepatic miR-122, is a significant regulator of pro-fibrotic mediator production. It is made up of stable HIF-1β subunit and a labile HIF-1α subunit. Previous report showed that HIF-1α was able to boost the expressions of vascular endothelial growth factor, monocyte chemoattractant protein-1, connective tissue growth factor, lysyloxidase-1, and platelet derived growth factor, which all play a significant role in fibrogenesis^3^.

Nucleotide-binding oligomerization domain-like receptor 3 (NLRP3) is an inflammasome upregulated and activated in hepatic injury, which further enhance the level of inflammatory cytokines^4^,^5^. The boosted level of inflammatory cytokines is responsible for the stimulation and multiplication of hepatic stellate cells. However, the inactivation of NLRP3 may protect hepatic injury resulting from excessive free fatty acids (FFAs)^6^,^7^.

AMP-activated protein kinase (AMPK) is considered an energy regulator that preserves lipid metabolism stability and is strongly related to the whole course of NAFLDs onset and progression. Previous reports suggested that AMP/ATP ratio activates AMPK, which leads to reducing the hepatic synthesis of fat, elevating the oxidation of fatty acid and boosting mitochondrial function in adipose tissue^8^,^9^. Sirtuin 1 (SIRT1) is a protein deacetylase based on nicotinamide adenine dinucleotide (NAD+) level which maintains the balance of lipid metabolism and energy. The inhibition of SIRT1 suppresses the cellular activity and is related with deposition mitochondrial and lipid dysfunction. However, its activation leads to sterol regulatory element binding protein-1c (SREBP-1c) phosphorylation, that reduces hepatic fat synthesis, and boosts fat breakdown and the fatty acid oxidation. So, targeting SIRT1 and AMPK pathways activation is a way of NAFLD treatment^10^. Moreover, pharmacological activation of nuclear factor erythroid 2-related factor (Nrf2) signaling results in boosting the gene expression of NAD (P) H-quinone oxidoreductase 1 (NQO1) and heme oxygenase-1 (HO-1), which in turn have inhibitory effect on oxidative stress and inflammation. Reports suggested that reduction of excessive oxidative is a possible way to treat NAFLD^2^,^11^.

Hippocrates is the well-known founder of medicine, who studies the many medicinal belongings of food and concise food as medicine. Various traditional system of medicine such as the Chinese one are practicing the use of food as medicine throughout history. Lotus (Nelumbo nucifera) is an amazing aquatic plant which has a rich cultural and medicinal inheritance in China, Korea, and other central East human settlements. Its leaves and other parts of the plant have been used for centuries in traditional cooking and folk medicine^12^–^14^. The plant does not have only aesthetic value, but also a variety of bioactive molecules. It also contains polyphenols such as catechin and quercetin, in addition to several glycosides of quercetin, kaempferol, and myricetin, which bestow its strong medicinal properties^12^,^13^.

Rich investigations on lotus extracts have indicated a broad profile of pharmacological and physiological activities. Research has shown lotus’ ability to help maintain healthy blood sugar levels, protect cells from damage, ward off a bacterial infection, neutralize free radicals that attack the body’s nerves and organs, and help maintain healthy weight. These pleiotropic therapeutic activities have been attributed to the synergistic roles of its bioactive ingredients, which are not only found in the leaves, but also in the seeds and rhizomes of the herb^12^,^15^–^18^. A number of scientific evidences have indicated the potential bioactivities of lotus in protecting human health, renewing interest in this valuable plant as a natural source of functional and traditional food and in medicinal applications in modern times^13^,^15^.

The current investigation scrutinized the hepatoprotective effect of lotus leaf (LF) against high-fat diet (HFD) induced liver injury in rats and explored the underlying mechanism.

## Methods

### Animals

Thirty-six albino rats (male; weight 200 ± 50 g and aged: 10–12 weeks old) were used in this study. The rats were procured from the animal house and kept in single polyethylene cages, in standard laboratory conditions (temperature 22 ± 5°C, relative humidity 60–70% and 12/12-h dark/light cycle). The rats received water ad libitum and standard food pellet. This study was approved by Medical Ethics Committee of Hebei Provincial Hospital of Traditional Chinese Medicine (Approval No.: HBZY2024-KY-091-01).

The experimental study was approved by the Institutional Animal Care and Use Committee. The research was conducted in the Department of Hepatobiliary Medicine, Medical Ethics Committee of Hebei Provincial Hospital of Traditional Chinese Medicine, China, from January 2025 to March 2025.

### Preparation of lotus leaf extract

The leaves of Nelumbo nucifera (lotus) was collected and shade dried. The shade dried leaves ground into a course powder. This course powder (200 g) was mixed with 4 L of ddH2O at the boiling point for 10 min. The LF extract was prepared via air suction filtration and afterward dried. The filtrate was then concentrated using the rotary evaporator under the reduced pressure at 40–50°C to remove the solvent. The resulting semisolid extract was further dried using the desiccator to yield a dry crude extract. The final extract was weighted and kept at 4°C in an airtight container for further use.

### Experimental protocol

#### High-fat diet

The rats received the HFD throughout the experimental study. The ingredients of the HFD are mentioned in Table 1.

**Table 1:** List of the high-fat diet ingredients.

S. No	Ingredient	Quantity
1	Casein	33.11٪
2	Starch	15.21٪
3	Cystine	0.30%
4	Cellulose	5.00%
5	Dextrose	15.21%
6	Soybean oil	5.00٪
7	Vitamins	1.00٪
8	Minerals	5.00٪
9	Lard	20.00٪
10	Colin	0.17٪

#### Test drug

The test drug (LF) was given to the rats in oral suspension form. The oral suspension was prepared via dissolved drug in 1% suspension of carboxymethyl cellulose. The test drug was selected on the basis of previous reported literature^19^.

### Experimental group

The rats were divided into six groups, and each group contained 12 rats, as follow:

- Group I: normal control;
- Group II: HFD;
- Group III: HFD + LF (25 mg/kg);
- Group IV: HFD + LF (50 mg/kg);
- Group V: HFD + LF (100 mg/kg);
- Group VI: HFD + simvastatin (4 mg/kg).

Each group of rats received the dose via oral gavage once a day for four weeks. The food and water intake, and behavioral activity were observed at regular time intervals. The body weight was estimated at every four days up to the end of the experimental study.

### Estimation of liver injury

The liver injury was accessed on the basis of scored system according to the previous reported method with minor modification^20^. The severity of the liver injury was accessed according to the degree of necrosis, focal and coagulative central area. The degree of lesions was graded from 1 to 5 depending on severity:

- Score 0 (normality);
- Score 1 (minimal < 1%);
- Score 2 (slight 1–25%);
- Score 3 (moderate 26–50%);
- Score 4 (moderate/severe 51–75%);
- Score 5 (severe/high 76–100%).

The percentage shows the proportion of the injured area in the photographed area.

### Biochemical parameters

At the end of the study, serum insulin was estimated by the previous reported protocol^21^, and one touch system was used for the estimation of glucose level (Johnson & Johnson, United States of America). The homeostatic model assessment of insulin resistance (HOMA-IR)was estimated using Eq. 1 22:


$$\text{ HOMA IR }=\frac{\text{ Fasting Insulin }\times \text{ Fasting Glucose }}{405}$$ (1)

The hepatic parameters like aspartate aminotransferase (AST), alanine aminotransferase (ALT), alkaline phosphatase (ALP), and gamma-glutamyl transferase (γ-GT); antioxidant parameters such as glutathione (GSH), superoxide dismutase (SOD), catalase (CAT), glutathione peroxidase (GPx), and malondialdehyde (MDA); lipid parameters viz., total cholesterol (TC), triglycerides (TG), low-density lipoprotein (LDL), high-density lipoprotein (HDL), and very-low density lipoprotein (VLDL); inflammatory cytokines, including tumor necrosis factor-α (TNF-α), interleukin-1β (IL-1β), interleukin-6 (IL-6), interleukin-10 (IL-10), interleukin-17 (IL-17), and interleukin-33 (IL-33); and HO-1 and Nfr2 were measured using the enzyme-linked immunosorbent assay (ELISA) kits, following the manufacture’s instruction.

### RNA isolation and quantification

Trizol reagent was used for the isolation of total RNA from the harvested liver tissue using the kits (Life Technologies, Inc., Grand Island, NY, United States of America). Then, the reverse transcription from total RNA to cDNA was processed via high-capacity cDNA reverse transcription kit (Applied Biosystems, Carlsbad, CA, United States of America) following the manufacture’s instruction. Concisely, RNA was diluted to reverse transcriptase (RT) master mix buffer 1 µL RNase inhibitor, 1 µL hexamer primer and 2 µL dNTP (10 mM) of diluted RNA on ice. Before adding 2 µL of RT (200 unit/µL), the mixture was heated for 10 min at 65°C and then snap cooled on ice for 2 min. After that, the reaction was performed for 10 min at 25°C, 120 min at 37°C, and 72°C for 4 min. Finally, the cDNA was kept at 80°C for further use.

### Liver tissue preparation

The liver tissue was isolated in the all-group rats, washed in cold saline buffer, dried in the ashless filter paper, and weighed. The hepatic index was estimated via the ratio of the rat’s liver weight × 100. The liver was homogenized (10% w/v) in ice cold Tris HCl buffer (0.1 M; pH = 7.4). Finally, the homogenate was centrifuged at 3,000 rpm at 4°C for 15 min.

### Histopathological study

The rats were sacrificed, and the liver tissue was isolated immediately and fixed in the buffered formalin (10%), dehydrated in gradual ethanol (50–100%), and cleared using the xylene embedded in paraffin. The liver tissues (5 µm) were prepared and stained with hematoxylin-eosin dye following the standard procedure for photomicroscopic observations.

### Statistical analysis

The results of the study are expressed as mean ± standard deviation via GraphPad Prism version 8 (St Louis, United States of America). Statistically significant effect of drug treatment was analyzed using the one-way analysis of variance (ANOVA) followed by Dunnett’s t test when ANOVA was significant. A *p* < 0.05 was considered significant.

## Results

### Effect on insulin, glucose and homeostatic model assessment of insulin resistance

**Figure 1:**
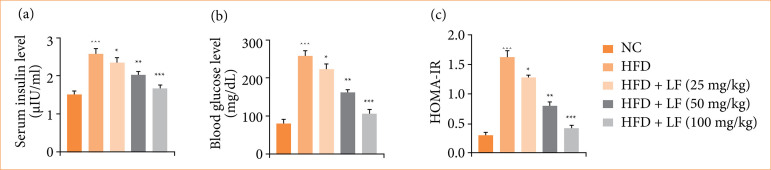
Effect of lotus (*Nelumbo nucifera*) leaf extract on the **(a)** serum insulin, **(b)** blood glucose level and **(c)** HOMAIR against non-alcoholic fatty liver disease in rats. All data are presented as mean ± standard error of the mean.

NC: normal control; HFD: high fat diet; LF: lotus leaf; HOMA-IR: homeostatic model assessment of insulin resistance; ###*p* < 0.001 was compared with the normal control; *p < 0.05 was compared with the HFD control group rats; **p < 0.01 was compared with the HFD control group rats; ***p < 0.001 was compared with the HFD control group rats. Source: Elaborated by the authors.

HFD group rats exhibited the enhancement in insulin (Fig. 1a), glucose level (Fig. 1b), and HOMA-IR (Fig. 1c), and LF treatment significantly (*p* < 0.001) reduced their level.

### Organ index

**Figure 2:**
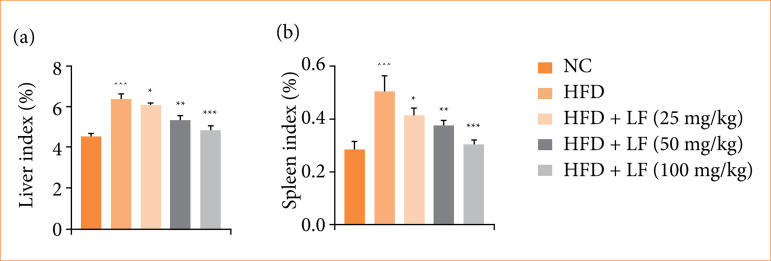
Effect of lotus (*Nelumbo nucifera*) leaf extract on **(a)** the liver index and **(b)** spleen index against non-alcoholic fatty liver disease in rats. All data are presented as mean ± standard error of the mean.

NC: normal control; HFD: high fat diet; LF: lotus leaf; ###*p* < 0.001 was compared with the normal control; *p < 0.05 was compared with the HFD control group rats; **p < 0.01 was compared with the HFD control group rats; ***p < 0.001 was compared with the HFD control group rats. Source: Elaborated by the authors.

HFD induced group rats exhibited increased liver index (Fig. 2a), and spleen index (Fig. 2b), and LF treatment significantly (*p* < 0.001) reduced the organ index.

### Hepatic parameters

**Figure 3:**
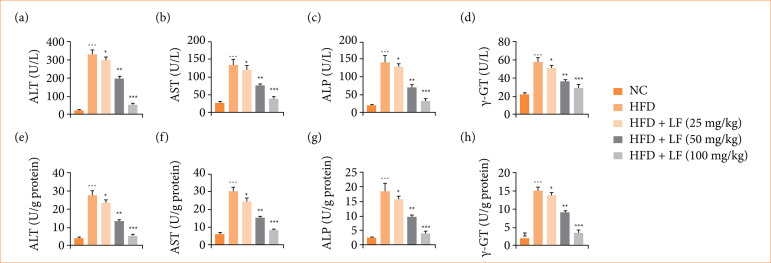
Effect of lotus (*Nelumbo nucifera*) leaf extract on the level of hepatic parameters against non-alcoholic fatty liver disease in rats. **(a)** alanine aminotransferase (ALT) in serum, **(b)** aspartate aminotransferase (AST) in serum, **(c)** alkaline phosphatase (ALP) in serum, **(d)** gamma-glutamyl transferase (γ-GT) in serum, **(e)** ALT, **(f)** AST, **(g)** ALP, and **(h)** γ-GT. All data are presented as mean ± standard error of the mean.

NC: normal control; HFD: high fat diet; LF: lotus leaf; ###*p* < 0.001 was compared with the normal control; *p < 0.05 was compared with the HFD control group rats; **p < 0.01 was compared with the HFD control group rats; ***p < 0.001 was compared with the HFD control group rats. Source: Elaborated by the authors.

HFD induced group rats demonstrated downregulation in the level of hepatic parameters (Fig. 3), including ALT, AST, ALP, and γ-GT, and LF treatment significantly (*p* < 0.001) reduced the level of hepatic parameters.

### Oxidative stress parameters

**Figure 4:**
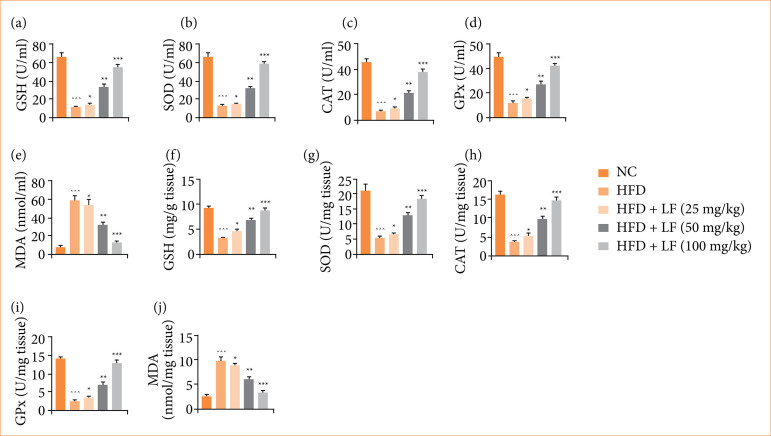
Effect of lotus (*Nelumbo nucifera*) leaf extract on the level of antioxidant parameters against non-alcoholic fatty liver disease in rats. **(a)** glutathione (GSH) in serum, **(b)** superoxide dismutase (SOD) in serum, **(c)** catalase (CAT) in serum, **(d)** glutathione peroxidase (GPx) in serum, **(e)** malonaldehyde (MDA) in serum, **(f)** GSH in tissue, **(g)** SOD in tissue, **(h)** CAT in tissue, **(i)** GPx in tissue, and **(j)** MDA in tissue. All data are presented as mean ± standard error of the mean.

NC: normal control; HFD: high fat diet; LF: lotus leaf; ###*p* < 0.001 was compared with the normal control; *p < 0.05 was compared with the HFD control group rats; **p < 0.01 was compared with the HFD control group rats; ***p < 0.001 was compared with the HFD control group rats. Source: Elaborated by the authors.

HFD induced group rats demonstrated altered level of oxidative stress parameters (Fig. 4), such as GSH, SOD, CAT, GPx, and MDA, and LF treatment significantly (*p* < 0.001) modulated the level of oxidative stress parameters.

### Lipid parameters

**Figure 5:**
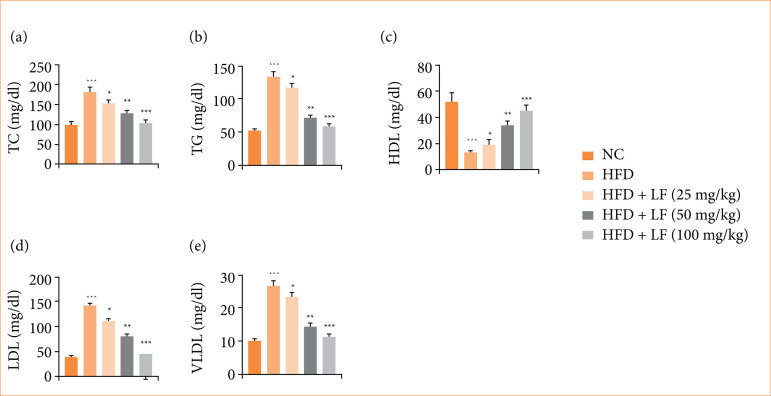
Effect of lotus (Nelumbo nucifera) leaf extract on the level of lipid parameters against non-alcoholic fatty liver disease in rats. **(a)** total cholesterol (TC), **(b)** triglyceride (TG), **(c)** high density lipoprotein (HDL), **(d)** low density lipoprotein (LDL), **(e)** very low-density lipoprotein (VLDL). All data are presented as mean ± standard error of the mean.

NC: normal control; HFD: high fat diet; LF: lotus leaf; ###*p* < 0.001 was compared with the normal control; *p < 0.05 was compared with the HFD control group rats; **p < 0.01 was compared with the HFD control group rats; ***p < 0.001 was compared with the HFD control group rats. Source: Elaborated by the authors.

HFD induced group rats revealed the altered level of lipid parameters such as TC (Fig. 5a), TG (Fig. 5b), HDL (Fig. 5c), LDL (Fig. 5d), and VLDL (Fig. 5e), and LF treatment significantly (*p* < 0.001) modulated the level of lipid parameters.

### Fat

**Figure 6:**
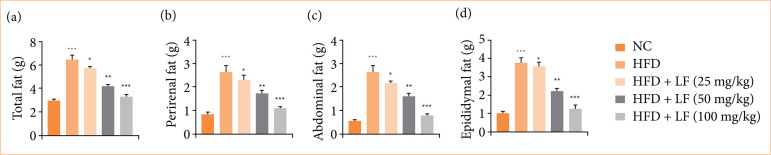
Effect of lotus (*Nelumbo nucifera*) leaf extract on the level of fat parameters against non-alcoholic fatty liver disease in rats. **(a)** Total fat, **(b)** perirenal fat, **(c)** abdominal fat, **(d)** epididymal fat. All data are presented as mean ± standard error of the mean.

NC: normal control; HFD: high fat diet; LF: lotus leaf; ###*p* < 0.001 was compared with the normal control; *p < 0.05 was compared with the HFD control group rats; **p < 0.01 was compared with the HFD control group rats; ***p < 0.001 was compared with the HFD control group rats. Source: Elaborated by the authors.

HFD induced group rats expressed the increased total fat (Fig. 6a), perirenal fat (Fig. 6b), abdominal fat (Fig. 6c), and epididymal fat (Fig. 6d), and LF treatment significantly (*p* < 0.001) decreased the level of fat related parameters.

### Cytokines

**Figure 7:**
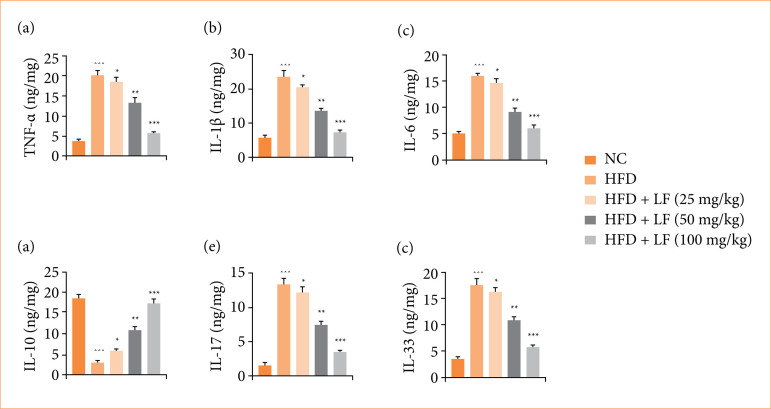
Effect of lotus (*Nelumbo nucifera*) leaf extract on the level of inflammatory cytokines parameters against non-alcoholic fatty liver disease in rats. **(a)** Tumor necrosis factor-α (TNF-α), **(b)** interleukin- (IL)-1β, **(c)** IL-6, **(d)** IL-17, **(e)** IL-33. All data are presented as mean ± standard error of the mean.

NC: normal control; HFD: high fat diet; LF: lotus leaf; ###*p* < 0.001 was compared with the normal control; *p < 0.05 was compared with the HFD control group rats; **p < 0.01 was compared with the HFD control group rats; ***p < 0.001 was compared with the HFD control group rats. Source: Elaborated by the authors.

HFD induced group rats expressed altered level of the cytokines TNF-α (Fig. 7a), IL-1β (Fig. 7b), IL-6 (Fig. 7c), IL-10 (Fig. 7d), IL-17 (Fig. 7e), and IL-33 (Fig. 7f), and LF treatment significantly (*p* < 0.001) modulated the level of cytokines.

### HO-1 and Nrf2

**Figure 8:**
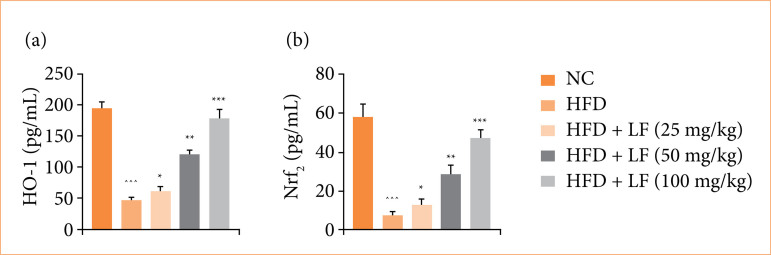
Effect of lotus (*Nelumbo nucifera*) leaf extract on the level of **(a)** heme oxygenase 1 (HO-1) and **(b)** nuclear factor erythroid 2-related factor (Nrf_2_) against non-alcoholic fatty liver disease in rats. All data are presented as mean ± standard error of the mean.

NC: normal control; HFD: high fat diet; LF: lotus leaf; ###*p* < 0.001 was compared with the normal control; *p < 0.05 was compared with the HFD control group rats; **p < 0.01 was compared with the HFD control group rats; ***p < 0.001 was compared with the HFD control group rats. Source: Elaborated by the authors.

HFD induced group rats expressed the suppressed levels of HO-1 (Fig. 8a), and Nrf2 (Fig. 8b), and LF treatment significantly (*p* < 0.001) enhanced the level of both.

**Figure 9:**
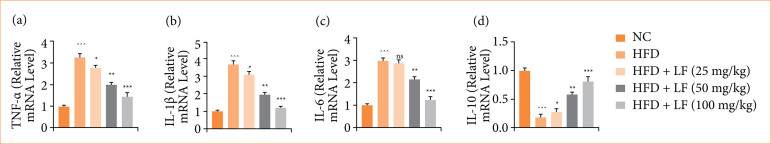
Effect of lotus (*Nelumbo nucifera*) leaf extract on the mRNA expression of inflammatory cytokines parameters against non-alcoholic fatty liver disease in rats. **(a)** Tumor necrosis factor-α (TNF-α), **(b)** interleukin- (IL)-1β, **(c)** IL-6, **(d)** IL-10. All data are presented as mean ± standard error of the mean.

NC: normal control; HFD: high fat diet; LF: lotus leaf; ###*p* < 0.001 was compared with the normal control; *p < 0.05 was compared with the HFD control group rats; **p < 0.01 was compared with the HFD control group rats; ***p < 0.001 was compared with the HFD control group rats. Source: Elaborated by the authors.

The inflammatory cytokines mRNA expression such as TNF-α (Fig. 9a), IL-1β (Fig. 9b), IL-6 (Fig. 9c), and IL-10 (Fig. 9d) were altered in HFD group rats, and LF treatment significantly (*p* < 0.001) modulated the expression.

**Figure 10:**
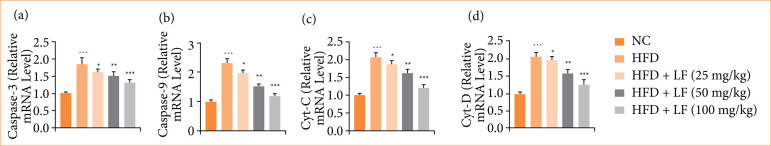
Effect of lotus (*Nelumbo nucifera*) leaf extract on the mRNA of **(a)** caspase-3, **(b)** caspase-9, **(c)** Cyt-C and **(d)** Cyt-D against non-alcoholic fatty liver disease in rats. All data are presented as mean ± standard deviation of the mean.

NC: normal control; HFD: high fat diet; LF: lotus leaf; ###*p* < 0.001 was compared with the normal control; *p < 0.05 was compared with the HFD control group rats; **p < 0.01 was compared with the HFD control group rats; ***p < 0.001 was compared with the HFD control group rats. Source: Elaborated by the authors.

HFD induced group rats exhibited the boosted mRNA expression of caspase-3 (Fig. 10a), caspase-9 (Fig. 10b), Cyt-C (Fig. 10c), and Cyt-D (Fig. 10d), and LF treatment significantly (*p* < 0.001) reduced the expression.

**Figure 11:**
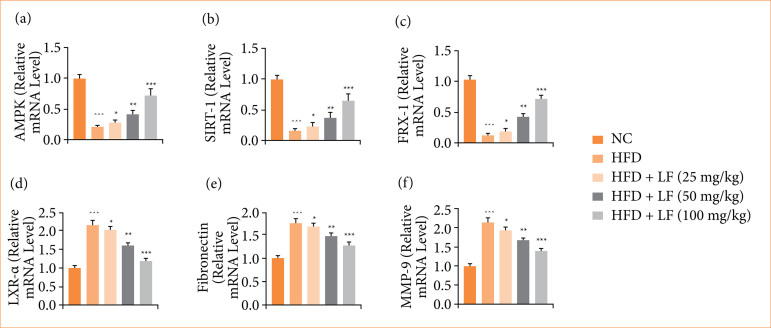
Effect of lotus (*Nelumbo nucifera*) leaf extract on the mRNA expression of **(a)** AMP-activated protein kinase (AMPK), **(b)** sirtuin 1 (SIRT-1), **(c)** FBX-1, **(d)** liver X receptor alpha (LXR-α), **(e)** fibronectin and **(f)** matrix metalloproteinase-9 (MMP-9) against non-alcoholic fatty liver disease in rats. All data are presented as mean ± standard error of the mean.

NC: normal control; HFD: high fat diet; LF: lotus leaf; ###*p* < 0.001 was compared with the normal control; *p < 0.05 was compared with the HFD control group rats; **p < 0.01 was compared with the HFD control group rats; ***p < 0.001 was compared with the HFD control group rats. Source: Elaborated by the authors.

HFD induced group rats expressed reduced mRNA expression of AMPK (Fig. 11a), SIRT-1 (Fig. 11b), FRX-1 (Fig. 11c), and increased mRNA expression of LXR-α (Fig. 11d), fibronectin (Fig. 11e), and MMP-9 (Fig. 11f). LF treatment significantly (*p* < 0.001) altered the mRNA expression.

**Figure 12:**
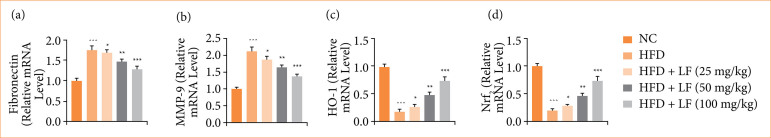
Effect of lotus (*Nelumbo nucifera*) leaf extract on the mRNA expression of **(a)** inducible nitric oxide synthase (iNOS), **(b)** transforming growth factor-β1 (TGF-β1), **(c)** heme oxygenase-1 (HO-1), and **(d)** nuclear factor erythroid 2-related factor 2 (Nrf_2_) against non-alcoholic fatty liver disease in rats. All data are presented as mean ± standard error of the mean.

NC: normal control; HFD: high fat diet; LF: lotus leaf; ###*p* < 0.001 was compared with the normal control; *p < 0.05 was compared with the HFD control group rats; **p < 0.01 was compared with the HFD control group rats; ***p < 0.001 was compared with the HFD control group rats. Source: Elaborated by the authors.

HFD induced group rats showed the boosted mRNA expression of inducible nitric oxide synthase (iNOS) (Fig. 12a), and transforming growth factor-β1 (TGF-β1) (Fig. 12b), and LF treatment significantly (*p* < 0.001) reduced the mRNA expression.

HFD group rats also exhibited the suppressed mRNA expression of HO-1 (Fig. 12c), and Nrf2 (Fig. 12d), and LF treatment improved the mRNA expression.

### Histopathology

**Figure 13:**
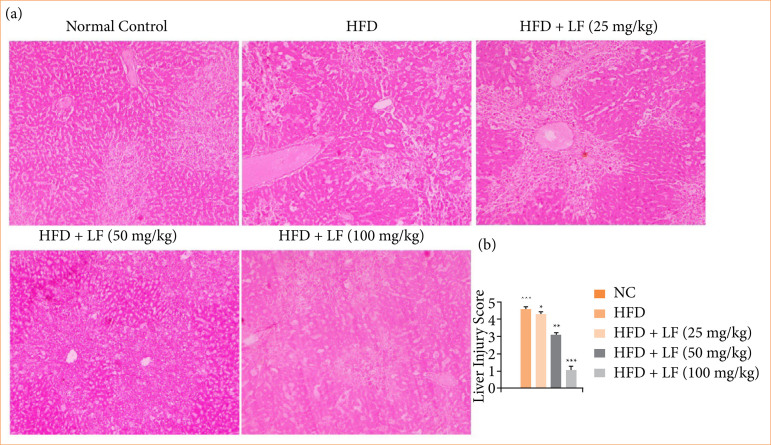
Effect of lotus (*Nelumbo nucifera*) leaf extract on **(a)** the liver histopathology and **(b)** liver injury against non-alcoholic fatty liver disease in rats. All data are presented as mean ± standard error of the mean.

NC: normal control; HFD: high fat diet; LF: lotus leaf; ###*p* < 0.001 was compared with the normal control; *p < 0.05 was compared with the HFD control group rats; **p < 0.01 was compared with the HFD control group rats; ***p < 0.001 was compared with the HFD control group rats. Source: Elaborated by the authors.

Normal group rats’ liver showed normal architecture with clear sinusoidal space, minimal or no inflammatory infiltrates. HFD group rats exhibited the disruption of hepatic architecture degeneration, necrosis of hepatocytes, inflammatory cell infiltration, Kupffer cell proliferation and activation. Dose dependently treatment of LF reduced the necrosis of hepatocytes with less inflammatory cell infiltration (Fig. 13a).

Normal group rats exhibited no score of liver injury, but HFD group rats exhibited increased liver injury score, which was dose-dependently suppressed by the LF treatment (Fig. 13b).

## Discussion

NAFLD is the most common cause of liver disease globally^23^. The most important role of the NAFLD pathogenesis is the dysfunction in hepatic cells, and it accelerates hepatic de novo lipogenesis, and increases the degree of insuline resistance (IR), resulting in a vicious cycle. The imbalance between lysis, lipid synthesis and absorption is caused by impairment insulin signaling, which increases lipolysis in adipocytes and increases the hepatic FFA input, thus leading to FFA buildup in hepatocytes that later contribute to hepatic steatosis from steatosis into HCC, NASH, and cirrhosis^2^,^23^,^24^. Drugs are commonly used in this disease treatment and have been developed intensely and quickly. The aim of the current study was to scrutinize the hepatoprotective effect of LF against HFD induced hepatic injury.

Fructose is an edible sugar commonly found in fruits and honey. Using fructose instead of granulated sugar in daily diet could decrease caloric intake with the same sweetness, and its glycemic index is very low^2^,^25^. Last few decades, excessive intake of fructose with diet has greatly increased. Liver is the main organ for utilization of fructose and its metabolism. Report suggested that high intake of fructose may be a significant risk factor for liver injury^25^.

HFD is an extremely high-risk factor for body weight and obesity gain. This association is strongly supported in the scientific literature, and demonstrates the importance of diet for general health^26^. Both obesity and NAFLD are associated with enhanced adipose tissue mass, hypertrophy, and hyperplasia of adipocytes. ATP has an essential role in mass and energy balance coordination to metabolic demand of the organism.

NAFLD is characterized by the accumulation of fat in the liver cells or hepatocytes. The disease spectrum begins with the more benign non-alcoholic fatty liver and progresses to the more aggressive metabolic dysfunction associated with steatohepatitis (MASH)^27^,^28^. MASH is characterized by the liver and fatty liver inflammation. When untreated, these chronic diseases can result in life-threatening complications such as cirrhosis, fibrosis, liver failure, or liver cancer. NAFL, the first stage of NAFLD, is characterized by the accumulation of fat in the liver with unknown cause such as consuming alcohol in large quantity. Diagnosis usually needs 5% or more of hepatocytes to contain visible lipid. The absence of symptoms in typical cases, however, tends to obscure the diagnosis, which emphasizes the importance of enhanced investigation and detection^28^,^29^.

Following HFD treatment, animals showed a robust increase in lipid profile compared with the control group rats, suggesting the establishment of dyslipidemia. It is defined by abnormal amounts of various lipids in the blood–HDL-C, TG, TC, and LDL-C. One of the major risk factors for atherosclerosis, a progressive disorder involving the build-up of plaque in the walls of the arteries, causing restricted blood flow and potential cardiovascular events, is dyslipidemia^30^–^32^.

The results of the present study showed that LDL deposition in intima-media thickness (IMT) as an LDL probe was greatly evident in HFD-fed animals. The intima-media constitutes the inner layer of the vascular wall, and its thickening is an early marker of atherosclerosis. This accumulation of LDL in the IMT indicates that the hyperlipidemic diet influence not only the systemic lipid but also vascular structure and health^32^,^33^. These changes in the vessel wall may also promote further progression of atherosclerosis, ultimately resulting in the development of plaques, vessel closure and an elevated risk of cardiovascular events, such as heart attack, and stroke^26^.

Reports suggested that oxidative stress dysfunction and systemic inflammation have been considered as a conjoint pathological mechanism, which play a crucial role in the progression and initiation of liver injury^23^,^34^. Generally, oxidative stress refers to an imbalanced state between antioxidant and oxidative activity within the body. Oxygen is decreased via electrons as part of normal metabolism leading to the excessive deposition of numerous reactive oxygen species (ROS). ROS is well known for their role in mediating both pathophysiological and physiological signal transduction^35^,^36^. Excessive production of ROS induces the oxidative injury to lipid peroxidative, cellular components, degradation of gastric epithelial membrane base, damage of cell membranes, and finally DNA damage. Activation of Kupffer cells, specialized macrophages in the liver, in response to damage is responsible for liver’s repair. These activated Kupffer cells also promote a higher level of oxidative stress and the release of inflammatory mediators^20^. These chemokines activate neutrophils, and other inflammatory cells, thus augmenting liver damage. Inflammation is a reaction that is regulated by numerous transcription factors, such as NF-κB, which mediates the expression of multiple inflammation-related genes^24^,^35^,^36^. Furthermore, inflammation is linked to the catalysis of two major inflammatory mediators: iNOS and COX-2. Nitric oxide produced by iNOS can react with superoxide free radicals to produce peroxynitrite, a mediator of free radical toxicity. The role of COX-2 in the pathogenesis of liver diseases is also complicated. This cross-talk between oxidative stress and inflammation forms a cycle, which when uncontrolled, can result in chronic liver insults^36^,^37^.

Nrf2 signaling is a master regulator of cellular oxidative status. It is a transcription factor that regulates the cellular antioxidant response, as well as redox homeostasis, by inducing an array of antioxidant genes. HO-1, one of such genes, is a vital antioxidant enzyme and catalyzes the degradation of heme to ferrous iron, carbon monoxide, and biliverdin^38^,^39^.

The prophylactic role of Nrf_2_ activation has been gaining appealing interest in the natural products research. Indeed, phytochemicals have been found to be able to activate Nrf2 antioxidant signaling for cytoprotective benefits^39^,^40^. This cumulative evidence indicates that natural products could be regarded as potential therapeutic agents for the prophylaxis of drug-induced hepatic injuries by tailoring oxidative stress mechanisms. The crosstalk among activation of Nrf2, induction of antioxidant genes, and the protective effects of natural products is an interesting area to pursue in hepatoprotection and drug toxicity control^38^,^41^.

AMPK acts as a fuel gauge that preserves cellular energy homeostasis. Sensitivity of this kinase to AMP:ATP ratios is high, such that it becomes active during energy stress and activates metabolic pathways involved in restoring the AMP vs. ATP balance^25^,^42^. The development of novel research has broadened our knowledge about the protective effects and molecular regulation of AMPK; one novel and important role is the regulation of Nrf_2_/HO-1 related signaling, the main defense response to oxidative stress and inflammation within cells in recent decades^43^,^44^. The crosstalk between AMPK and the Nrf_2_/HO-1 pathway constitutes a classic crosstalk between energy metabolism and antioxidation. Upon activation of AMPK, the activation of the Nrf_2_/HO-1 pathway is stimulated, thereby promoting the transcription of cytoprotective genes and enzymes. Such interplay among pathways that regulate the cellular redox state, energy metabolism, and protein homeostasis further emphasizes the complexity and interconnectedness of cellular networks^44^. The interlink between AMPK and Nrf_2_/HO-1 signalling and its potential in treatment of metabolic disease and cancer resurge in the new clinical perspective as a therapeutic option, particularly the cross-activation of these pathways that can control oxidative stress, inflammatory status, and cell metabolism, conferring protective actions against the progression of diseases and improving treatment responses^42^.

The favorable role of AMPK activation on hepatic steatosis, an abnormal accumulation of fat in the liver, has been demonstrated. This results in the phosphorylation of acetyl Co-A carboxylase (ACC), a rate limiting enzyme of lipogenesis, and specifically induction in the activity of ACC oxidase, an enzyme of fatty acid oxidation^8^,^45^. The action mechanism of LF is through the up expression of peroxisome proliferator-activated receptor γ coativator 1α (PGC1α), increased ratio of p-AMPK/AMPK, and depress expression of sterol regulatory element binding protein 1c (SREBP-1c) and FA synthetase (FAS). This is due to the inhibition of mitochondrial respiratory chain complex I and an increase in the ratio between AMP and ATP content, which causes activation of AMPK by phosphorylation on the Thr172 sports. Phosphorylated AMPK is a pleiotropic signaling executor. It inhibits the activity of the enzyme ACC, that is responsible for lipid synthesis, but it increases the level of CPT1 and PGC1α^45^,^46^. These alterations result in enhanced fatty acid oxidative and suppressed lipid synthesis. The latter conclusion is further supported by the decrease of SREBP-1c and its target proteins (e.g., FAS). Furthermore, the hepatic expression of nuclear transcription factors, including FXR and LXR and regulators of hepatic lipogenesis, is altered. Lipid-modulatory effects of AMPK taken together, this broad range of lipid-modulatory activities suggests that AMPK activation could be a potential therapeutic target in hepatic steatosis and associated metabolic disorders^47^,^48^.

## Conclusion

The LF altered the expression of critical genes related to apoptosis regulation, and lipid metabolism, and inflammation was affected by LF extract. These protective effects may be attributed to, at least partly, the stimulating effects on the AMPK/SIRT1 and Nrf2/HO-1 signaling pathways and suggest the LF potential application in the treatment of NAFLD.

## Data Availability

The data will be available on the request to the corresponding author.
